# Intravital Imaging Reveals Dynamics of Lymphangiogenesis and Valvulogenesis

**DOI:** 10.1038/srep19459

**Published:** 2016-01-20

**Authors:** Gyeong Jin Kang, Tatiana Ecoiffier, Tan Truong, Don Yuen, Guangyu Li, Narae Lee, Liwei Zhang, Lu Chen

**Affiliations:** 1 Vision Science Graduate Group, University of California, Berkeley, CA 94720, USA; 2 Center for Eye Disease and Development, Program in Vision Science, and School of Optometry, University of California, Berkeley, CA 94720, USA

## Abstract

Lymphatic research signifies a field of rapid progression in recent years. Though lymphatic dysfunction has been found in a myriad of disorders, to date, few effective treatments are available for lymphatic diseases. It is therefore urgent to develop new experimental approaches and therapeutic protocols. The cornea offers an ideal site for lymphatic research due to its transparent nature, accessible location, and lymphatic-free but –inducible features. Moreover, we have recently discovered that corneal lymphatic vessels develop luminal valves as lymphangiogenesis proceeds. This tissue thus provides an optimal tool to study both lymphangiogenesis and valvulogenesis upon a pathological insult. In this paper, we show that the modified Prox-1-GFP mice carrying wildtype C57BL/6 background provide a valuable tool for intravital imaging of corneal lymphatic vessels and valves and can be used to study pathological lymphangiogenesis induced by various insults. Further, we demonstrate the multifaceted dynamics of lymphangiogenesis and valvulogenesis associated with transplantation, from the initiation to regression phases, and report several novel and critical phenomena and mechanisms that cannot be detected by conventional *ex vivo* approaches. Further investigation holds the great potential for divulging new mechanisms and therapeutic strategies for lymphangiogenesis and lymphangiogenesis-related diseases at various stages and inside or outside the eye.

The lymphatic system plays essential roles in immune surveillance, body fluid homeostasis, and dietary fat and vitamin absorption. Despite their importance, research on lymphatic vessels has been overshadowed for centuries compared to blood vessels due to their invisibility. It has been vitalized in recent years when several lymphatic specific markers, such as LYVE-1 (lymphatic vessel endothelial hyaluronic acid receptor-1) and Prox-1 (prospero homeobox protein-1; the master control gene for lymphatic development), were identified for lymphatic recognition. Lymphangiogenesis (LG), the growth of new lymphatic vessels, is critically involved in a wide array of diseases and pathological conditions, which include but are not limited to inflammation, infection, cancer metastasis, and transplant rejection^1^,^2^,^3^. However, up to this stage, there are still few effective treatments for lymphatic disorders. It is therefore both urgent and important to develop new experimental approaches and therapeutic strategies.

The cornea offers an ideal site for pathological LG research due to its accessible location, transparent nature, and alymphatic status under normal condition. Since there are no background lymphatics to consider, it is exceptionally straightforward and accurate to assess pathological LG in this tissue^2^. We have also recently discovered that corneal lymphatics develop luminal valves as lymphatic vessels mature and these valves play an important role in directing lymph flow^4^,^5^. Taken together, the cornea provides an optimal model to investigate pathologic events of both LG and valvulogenesis (VG, the formation of new lymphatic valves), and intravital imaging of dynamic lymphatic events in this tissue should have broad applications in biomedical research.

In this study, we report that Prox-1-GFP (green fluorescence protein) mice carrying a wildtype C57BL/6 background offer a valuable tool for intravital imaging of newly formed lymphatic vessels, as well as valves, within the cornea. They can be used to observe LG stimulated by a variety of pathological insults, such as suture-induced inflammation, growth factor implantation, and transplantation, and to evaluate the therapeutic effect of a pharmaceutical intervention. More importantly, by performing time course intravital imaging in these mice, we reveal for the first time the multifaceted temporal and spatial dynamics of LG and VG induced by corneal transplantation. Further, we show that the pathological lymphangiogenic event is an active and complex process from the initiation to regression phases and report several new phenomena and mechanisms that cannot be detected by conventional *ex vivo* study, including VG initiated from inside the limbal vessels, lymphatic elongation achieved by stalk cell lateral migration, lymphatic pruning from vessel tips, and lymphatic regression within late stage vessels. Taken together, our findings not only offer novel insights into pathological LG and VG, but also provide critical information for further investigation and modulation of LG and LG-related diseases at various stages.

## Results

### Corneal intravital imaging in modified Prox-1-GFP mice

**Figure 1:**
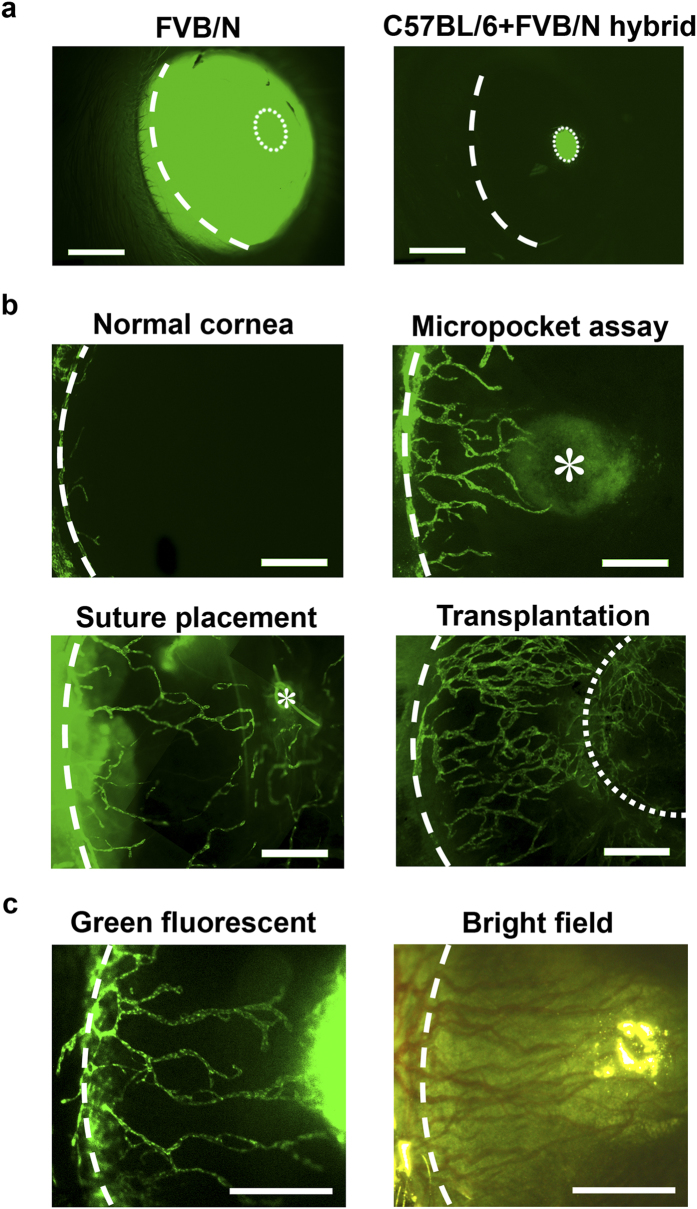
Corneal intravital imaging in modified Prox-1-GFP mice showing lymphatic vessels induced by various insults. (**a**) Intravial images of Prox-1-GFP mice of the FVB/N background and FVB/N-C57BL/6 hybrid showing high fluorescence signals from lens epithelium were blocked by the pigmented iris in the hybrid. Scale bars: 1 mm. (**b**) Intravital images demonstrating newly formed lymphatic vessels induced by various pathological insults. Scale bar: 500 μm. (**c**) Intravital images of the same cornea post-suturing showing newly formed lymphatic vessels (green) visualized under green fluorescent light and blood vessels (red) under bright field light. Scale bar: 500 μm. Dashed line: demarcation of the limbus between the cornea and conjunctiva; Dotted line: pupil margin (**a**) or graft-host border (**b**); Asterisk: site of VEGF-C pellet implantation or suture placement (**b**).

Prox-1-GFP mice in FVB/N background have been reported to faithfully recapitulate the expression of Prox-1 in lymphatic endothelial cells^6^. However, they are not suitable for live imaging in the cornea due to the constitutive expression of Prox-1 in lens epithelium. As shown in Fig. 1a, to enable intravital imaging of lymphangiogeneic process within the cornea, we cross-bred the founder Prox-1-GFP mice in FVB/N background with wildtype C57BL/6 mice. The highly pigmented iris in black C57BL/6 mice, an anatomical structure located between the cornea and lens, blocks lens interference and hence allows for corneal imaging.

### Intravital imaging visualizes lymphangiogenesis induced by various insults

We next demonstrated that the modified transgenic mouse model can be used to visualize corneal LG induced by various pathologic insults, including micropocket implantation of vascular endothelial growth factor-C (VEGF-C), suture placement, and transplantation (Fig. 1b). These are the most commonly used LG models with the cornea^2^,^7^. We also demonstrated that both lymphatic and blood vessels can be detected in the cornea under fluorescein isothiocyanate (FITC) green fluorescent excitation source or LED with bright field (Fig. 1c).

**Figure 2:**
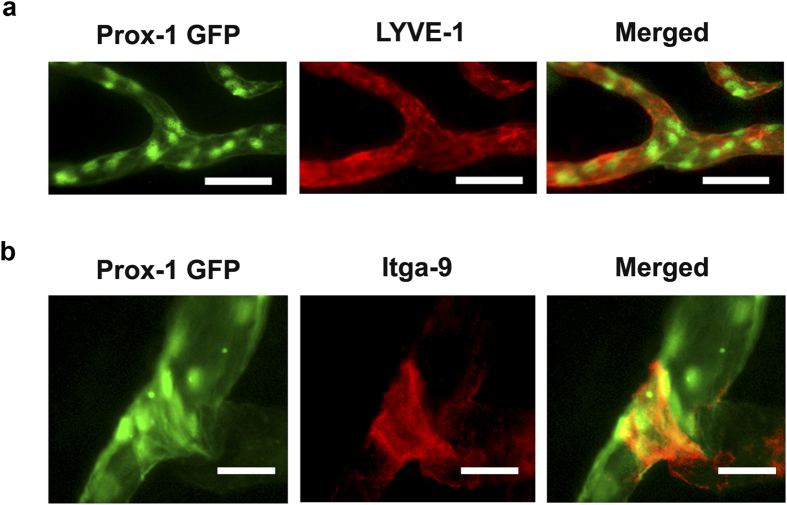
Immunofluorescent microscopic analysis confirming Prox-1 GFP signals identify lymphatic vessels and valves in the cornea. **(a)** Immunofluorescent microscopic images showing co-localization of Prox-1 GFP signal (green) and LYVE-1 staining (red) along newly formed lymphatic vessels in the inflamed cornea. Scale bars: 50 μm. **(b)** Immunofluorescent microscopic images showing co-localization of Prox-1 GFP signal (green) and Itga-9 staining (red) on the luminal valve of the lymphatic vessel. Scale bars: 25 μm.

To confirm the Prox-1 GFP positive vessels detected in the corneas of the transgenic hybrid mice were lymphatics, we performed immunofluorescent microscopic analysis using specific antibodies against LYVE-1, the most widely used marker for lymphatic identification. Our data verified the co-localization of Prox-1 GFP and LYVE-1 signals on corneal lymphatic vessels (Fig. 2a). Additionally, we also observed Prox-1 GFP signal on the luminal valves inside lymphatic vessels, and further immunofluorescent microscopic analysis validated their co-localization with integrin alpha-9 (Itga-9), the specific marker for lymphatic valves^4^ (Fig. 2b).

### Time course evaluation on pharmaceutical intervention on inflammatory lymphangiogenesis

**Figure 3:**
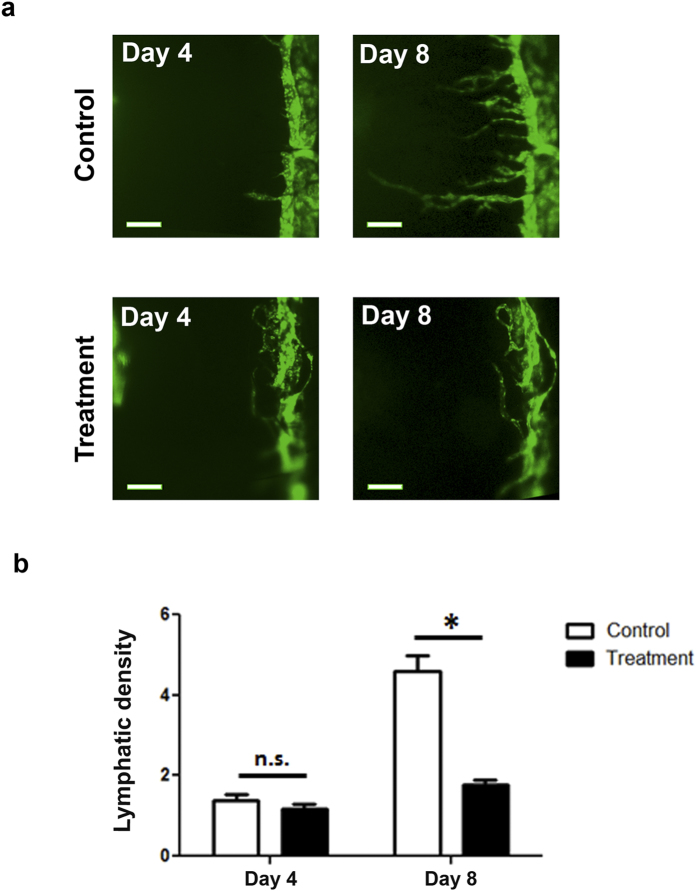
Intravital time course evaluation of a pharmaceutical intervention on inflammatory lymphangiogenesis. **(a)** Intravital images showing VEGFR-2 blockade via intraperitoneal injections of neutralizing antibodies suppressed corneal lymphatic vessel formation induced by suture placement. Scale bars: 200 μm. Summarized data are presented in **(b).** *P < 0.05.

To demonstrate the application of this mouse model for longitudinal investigation on LG and the effect of a pharmaceutical intervention, we performed a time course study and assessed the blockade effect of vascular endothelial growth factor receptor-2 (VEGFR-2) on inflammatory LG induced by suture placement^8^,^9^. As shown in Fig. 3a, a systemic blockade via intraperitoneal injection of VEGFR-2 neutralizing antibodies led to a significant suppression of inflammatory LG in the cornea. Summarized data from repetitive experiments are presented in Fig. 3b.

### Lymphatic valvulogenesis is initiated inside the limbal vessels

**Figure 4:**
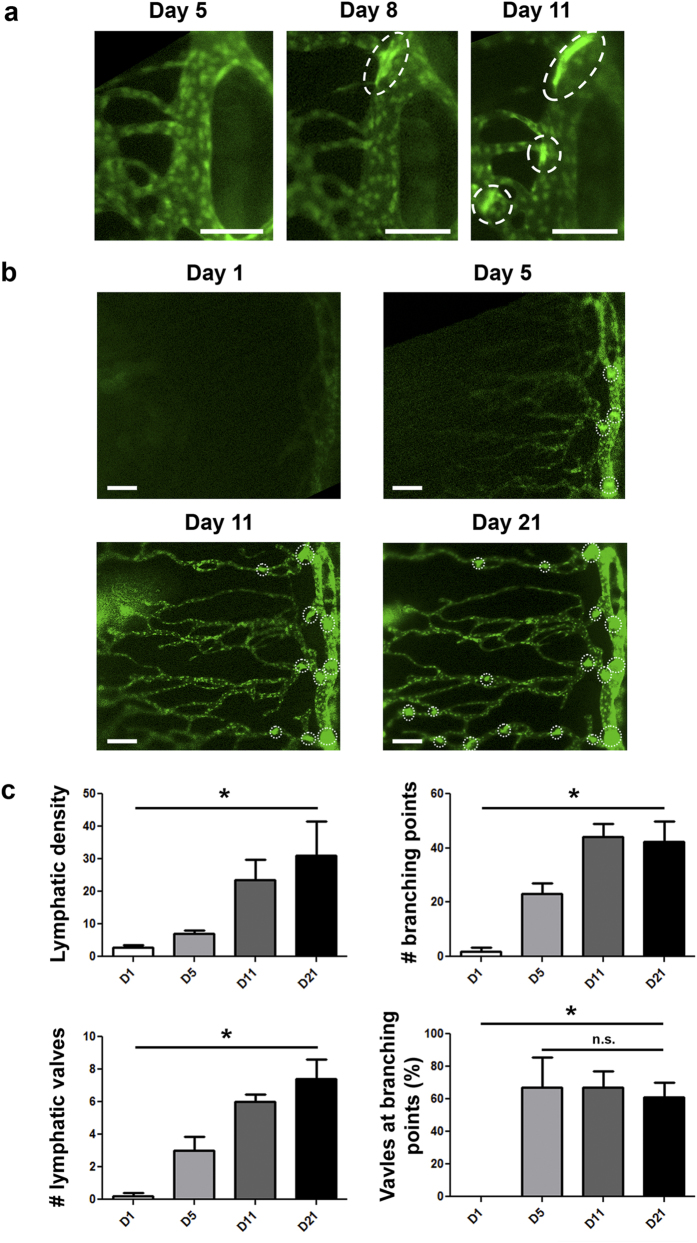
Intravital time course imaging showing the initiative and progressive processes of lymphangiogenesis and valvulogenesis after transplantation. (**a**) Intravital time course evaluation on the initiation and progression of valve formation within limbal vessels. Scale bars: 100 μm. (**b**) Intravital time course evaluation on progressive lymphangiogenesis in parallel with valvulogenesis into central cornea after transplantation. Scale bars: 150 μm. Vertical vessel to the right of the panel: limbal vessel; Dotted circles: newly formed valves. (**c**) Summarized data showing time course increase of lymphatic density, branching points, and valves. Lymphatic valves are predominantly located at the branching points across the time points studied with progressive LG and VG. *P < 0.05.

We next explored more details on the dynamics of the lymphangiogeneic event using the transplantation model, which vigorously induced both LG and VG, compared to other models (Figs 1 and 4). Surprisingly and interestingly, we found that VG was first initiated inside pre-existing limbal vessels after lymphatic sprouts budded from them. As shown in Fig. 4a, while only one valve was observed forming inside the limbal vessel on 8 days post transplantation, two more valves were detected within 3 days.

### Progressive valvulogenesis along with advancing lymphangiogenesis into central cornea

Following limbal vessel valve formation and along with LG marching into the central cornea, more valves were formed inside the elongating lymphatic vessels in time course (Fig. 4b). In the first week post transplantation, lymphatic sprouts were detected budding from limbal vessels indicating the early stage of LG. As the vessels advanced into the central cornea in the following 2 weeks, an increasing number of valves were observed inside these vessels as they extended. Summarized data reflecting the time course increases of lymphatic vessels and valves are presented in Fig. 4c. Moreover, we have also assessed the relationship between branching points and the location of lymphatic valves and found that across the time studied with progressive LG and VG, lymphatic valves were predominantly located near the branching points (Fig. 4c), and this result is in consistent with our previous one time point *ex vivo* study in the suture model^4^.

### Lymphatic endothelial stalk cell migration during vessel elongation

**Figure 5:**
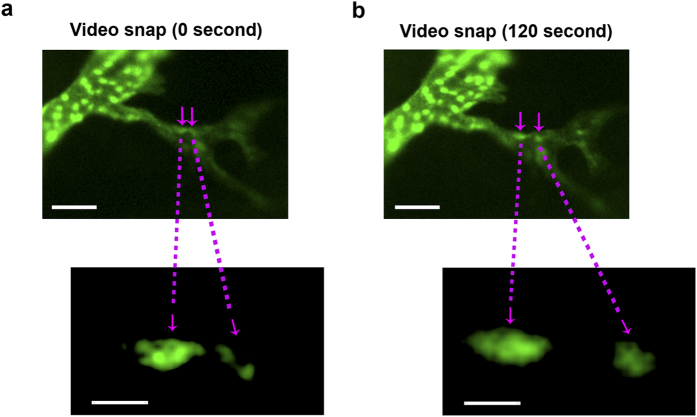
Intravital video showing lateral migration of stalk cells during lymphatic elongation. Snap pictures of intravital video illustrating departure of two endothelial stalk cells (indicated by magenta arrows) along the axis of vessel elongation. (**a**,**b**, top panels) Images showing the close proximity of the two cells before separation (**a**, 0 second) and after lateral migration (**b**, 120 seconds). Scale bars: 25 μm. Corresponding magnified images are presented in the lower panels. Scale bars: 5 μm.

Excitingly, with the live imaging technique, we were also able to divulge another important phenomenon and mechanism underlying lymphatic elongation, which is endothelial stalk cell lateral migration. As shown in Fig. 5, within a short period of 2 minutes, two stalk cells in close proximity had departed along the axis of vessel extension. This dynamic event occurring in the middle of a lymphatic branch is more vividly presented in Supplementary Video 1.

### Lymphatic pruning at early phase of lymphangiogenesis

**Figure 6:**
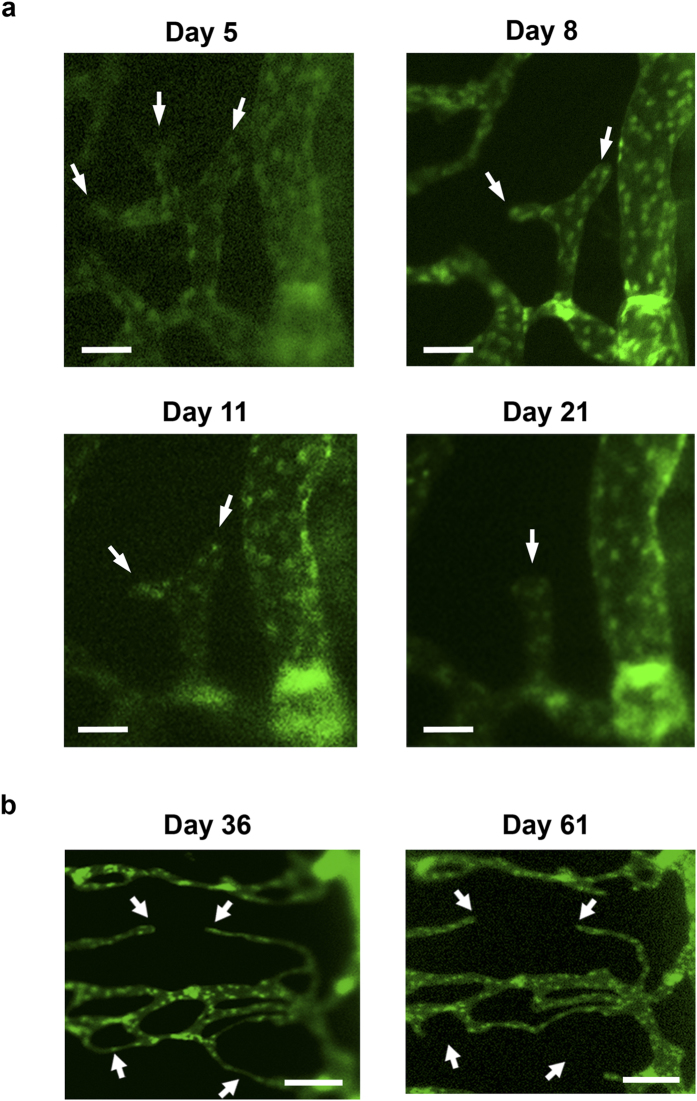
Intravital time course imaging demonstrating processes of lymphatic pruning and regression at the early and late stages after transplantation. (**a**) Representative intravital images showing a pruning process by tip retraction. Arrow: tip of lymphatic vessel. Scale bars: 50 μm. (**b**) Representative intravital images illustrating a regression process by breakage in the middle of the lymphatic branches. Arrow: site of breakage. Scale bars: 150 μm.

During the early phase of LG and within 3 weeks after transplantation, we also detected another novel phenomenon of lymphatic pruning. This appeared as a retraction of lymphatic fronts or tips of newly forming lymphatic capillaries while the other vessels were still growing and elongating (Fig. 6a).

### Lymphatic regression at late stage of lymphangiogenesis

In contrast, at the late stage of LG and after the lymphatic vessels reached to their maximal length around 3 weeks post transplantation, the process of lymphatic regression was observed with breakages in the middle of lymphatic branches, as illustrated in Fig. 6b.

## Discussion

In summary, this study using intravital imaging reveals novel aspects of the lymphangiogeneic events on both LG and VG in the cornea, one of the most favorite tissues for lymphatic research. These include progressive LG budding from the limbal vessels and marching into the central cornea, and subsequent valve formation and dissemination into the elongating lymphatic vessels after transplantation. In addition, we have divulged several novel phenomena involved in these dynamic events, such as the initiation of VG from inside limbal vessels, the spatial and temporal relationship between LG, lymphatic branching formation and VG, and distinctive patterns of lymphatic vessel elongation, pruning, and regression at different stages. We have exhibited as well the possibilities of using the live imaging model to examine blood vessels in parallel to lymphatic vessels and to evaluate the therapeutic effect of a pharmacological intervention.

Intravital imaging has great advantages over conventional immunohiscochemical method with dead tissues^8^. Without live imaging, it is impossible to track the dynamic changes of LG/VG in the same tissue or subject for a period of time and at the physiological status. This technical limitation has created enormous knowledge gaps in our understanding of the real processes engaged in pathological lymphangiogeneic events. In this study with the live imaging system, we have been able to track the same tissue and site over a short or long period of time and have obtained the first evidence revealing the dynamics of LG and VG induced by transplantation. The high quality intravital images and videos acquired over the course allow us to perform detailed temporal and spatial analysis on the vessels and valves at their natural status and this in-depth analysis leads to the discovery of several new and important phenomena that cannot be detected with conventional *ex vivo* methods.

Our finding that in the setting of pathological LG in adult mammalian tissue, lymphatic elongation is achieved by lateral migration of the stalk cells is both novel and important. It offers the first, direct, *in vivo* and in real time evidence on a critical cellular mechanism underlying pathological LG, which merits further investigation. It is speculated that lymphatic pruning may facilitate more efficient lymphatic flow by reducing the number of overall branches, similar to synaptic pruning^10^, which is yet to be explored. Our finding that lymphatic valves are first formed inside the pre-existing limbal vessels indicates that the limbal vessels not only give rise to lymphatic sprouts but also equip themselves with more valves for increased lymph flow in the diseased tissue. Collectively, pruning and valve formation may contribute to lymphatic maturation leaving functional vessels in network.

Research on corneal transplantation and LG is important because LG accompanies many diseases after inflammatory, chemical, infectious, immunogenic, or traumatic damage^2^, and it is a primary mediator of corneal transplant rejection^11^,^12^. LG-invaded corneas are hostile to transplants for vision restoration due to a high rejection rate reaching 50–90%, irrespective of current treatment modality^2^,^13^,^14^. It is thus crucial to investigate the dynamics of LG and to obtain real time information on when and how LG is initiated, progressed, remodeled, and regressed, which are prerequisites for developing new and effective therapeutic approaches for corneal graft rejection in the future.

Furthermore, corneal LG research has broader implications beyond the eye. The cornea has been used by many scientists in broad fields to investigate neo-vascular events for decades. It has been employed for tumor angiogenesis research since 1970s^15^ and more recently for LG research since 1990s^2^,^7^. Results from corneal vascular studies have also been proven to be readily applicable to other sites of the body. While a study using the corneal transplantation model has provided the first evidence on the importance of the lymphatic pathway in mediating graft rejection^16^, this concept has later been corroborated in other major organ transplantations as well^1^,^17^,^18^,^19^,^20^. Since the lymphatic network penetrates most tissues in the body and pathological LG has been associated with a wide array of disorders including cancer metastasis, and inflammatory and immune diseases^1^,^3^, it is plausible to predict that the lymphatic changes we observe in the cornea, whether in a short or long period of time, may also occur in other sites of the body. Results from this study may shed some light on our understanding and management of other LG-related disorders as well.

Taken together, we anticipate this study will facilitate lymphatic research in broad fields. Future investigation using the intravital imaging and on the novel phenomena promises for filling our knowledge gaps in understanding pathologic LG with VG and their management at various stages and inside or outside the eye.

## Methods

### Mice and Aanesthesia

All mice were treated according to ARVO Statement for the Use of Animals in Ophthalmic and Vision Research, and all protocols were approved by the Animal Care and Use Committee of the institutes. Prox-1 GFP hybrid mice were generated by cross breeding between wildtype C57BL/6 mice (Taconic Farms, Germantown, NY, USA) and Prox-1 GFP mice of FVB/N background^6^ (University of Southern California), and a total of 31 hybrid mice were used in the experiments unless otherwise indicated. Mice were anesthetized using a mixture of ketamine, xylazine, and acepromazine (50 mg, 10 mg, and 1 mg/kg body weight, respectively) for each surgical procedure.

### Corneal Suture Placement

The procedure was performed as previously reported^21^. Briefly, 11-0 nylon sutures

(AROSurgical, Newport Beach, CA) were placed intrastromally without penetrating into the anterior chamber.

### Corneal Micropocket Implantation

The procedure was performed as previously reported^22^. Corneal micropocket was created 1.0 mm apart from limbal vascular arcade using a modified von Graefe Knife and slow-release pellet was implanted into the pocket. The pellet was made of sucralfate (Sigma Aldrich, St. Louis, MO) and hydron polymer (Sigma Aldrich) containing 400 ng VEGF-C (R&D Systems, Minneapolis, MN) and left in place for 9 days.

### Corneal Transplantation

Standard orthotopic corneal transplantation was performed^12^. Briefly, the central cornea of the donor was marked with a 2-mm diameter micro-curette (Katena Products Inc., Denville, NJ) and excised with Vannas scissors (Storz Instruments Co, San Dimas, CA). The recipient graft bed was prepared by excising a circular 1.5-mm area in the central cornea. The donor button was placed onto the graft bed and secured with eight interrupted 11-0 nylon sutures (AROSurgical). Lymphatic density in the cornea from 11 to 1 o’ clock was graded and analyzed using the NIH Image J software^9^. The number of branching points and lymphatic valves from the same area were counted for each sample. The experiments were repeated twice with 5 mice in each group of the study.

### Intravital Imaging

The procedure was performed as we previously reported^5^,^8^. The samples were imaged with a customized imaging system including an adjustable eye and head stage holder and the Axio Zoom.V16 microscope (Carl Zeiss AG). Digital pictures were taken under FITC excitation light or LED bright field light. For Supplementary Video 1, series of time-lapse images were taken in a time frame of 1 image per 10 second. A total of 13 images taken within 120 seconds were streamed to 4 seconds with the NIH Image J software as reported previously^8^.

### Pharmaceutical Intervention

The experiments were performed as previously reported^9^. Mice after one suture placement at 12 o’ clock were randomized to receive intraperitoneal administration of VEGFR-2 neutralizing antibody (800 μg, DC101; Eli Lilly and Company, New York, NY) or PBS on Day 0 and Day 4 post-suturing. Lymphatic density in the cornea from 9 to 3 o’ clock area was graded and analyzed using the NIH Image J software^9^. Individual lymphatic vessels were highlighted and added together to generate a density score measured in pixels for each sample. The experiments were repeated twice with a total of 6 mice in the treatment and 7 mice in the control groups.

### Immunofluorescent Microscopic Assay

The experiments were performed as previously reported^4^,^21^. Briefly, whole-mount full thickness corneas were incubated with rabbit-anti-mouse LYVE-1 (Abcam, Cambridge, MA) or goat-anti-mouse Itga-9 antibodies (R&D Systems, Minneapolis, MN), and visualized by Cy3-conjugated donkey-anti-rabbit antibodies or Cy3-conjugated donkey-anti-goat antibodies (Jackson ImmunoResearch Laboratories, West Grove, PA), respectively. Samples were covered with Vector Shield mounting medium (Vector Laboratories, Burlingame, CA) and examined by an AxioImager M1 epifluorescence deconvolution microscope (Carl Zeiss AG, Göttingen, Germany).

### Statistical Analysis

The results are reported as mean ± SEM. Student *t*-test and one-way ANOVA were used to determine the significance levels between the groups for VEGFR-2 treatment experiment and transplantation experiment, respectively. The differences were considered statistically significant when *p* < 0.05 using Prism software (GraphPad, La Jolla, CA).

## Additional Information

**How to cite this article**: Kang, G. J. *et al*. Intravital Imaging Reveals Dynamics of Lymphangiogenesis and Valvulogenesis. *Sci. Rep*. **6**, 19459; doi: 10.1038/srep19459 (2016).

## Supplementary Material

Supplementary Information

Supplementary video S1

## References

[b1] Alitalo K. The lymphatic vasculature in disease. Nature medicine 17, 1371–1380 (2011). 10.1038/nm.2545 22064427

[b2] Chen L. Ocular lymphatics: state-of-the-art review. Lymphology 42, 66–76 (2009). 19725271 PMC4725303

[b3] Karaman S. & Detmar M. Mechanisms of lymphatic metastasis. The Journal of clinical investigation 124, 922–928 (2014). 24590277 10.1172/JCI71606 PMC3938272

[b4] Truong T., Altiok E., Yuen D., Ecoiffier T. & Chen L. Novel characterization of lymphatic valve formation during corneal inflammation. PloS one 6, e21918 (2011). 21760922 10.1371/journal.pone.0021918 PMC3131394

[b5] Truong T., Huang E., Yuen D. & Chen L. Corneal lymphatic valve formation in relation to lymphangiogenesis. Investigative ophthalmology & visual science 55, 1876–1883 (2014). 24595382 10.1167/iovs.13-12251 PMC3968927

[b6] Choi I. . Visualization of lymphatic vessels by Prox1-promoter directed GFP reporter in a bacterial artificial chromosome-based transgenic mouse. Blood 117, 362–365 (2011). 20962325 10.1182/blood-2010-07-298562 PMC3037757

[b7] Chen L., Hann B. & Wu L. Experimental models to study lymphatic and blood vascular metastasis. Journal of surgical oncology 103, 475–483 (2011). 21480239 10.1002/jso.21794 PMC3201795

[b8] Yuen D. . Live imaging of newly formed lymphatic vessels in the cornea. Cell research 21, 1745–1749 (2011). 22083511 10.1038/cr.2011.178 PMC3357992

[b9] Yuen D., Pytowski B. & Chen L. Combined blockade of VEGFR-2 and VEGFR-3 inhibits inflammatory lymphangiogenesis in early and middle stages. Investigative ophthalmology & visual science 52, 2593–2597 (2011). 21273538 10.1167/iovs.10-6408 PMC3088552

[b10] Zecevic N. & Rakic P. Synaptogenesis in monkey somatosensory cortex. Cerebral cortex 1, 510–523 (1991). 1822755 10.1093/cercor/1.6.510

[b11] Dietrich T. . Cutting edge: lymphatic vessels, not blood vessels, primarily mediate immune rejections after transplantation. Journal of immunology 184, 535–539 (2010). 10.4049/jimmunol.0903180 PMC4725297 20018627

[b12] Zhang H., Grimaldo S., Yuen D. & Chen L. Combined blockade of VEGFR-3 and VLA-1 markedly promotes high-risk corneal transplant survival. Investigative ophthalmology & visual science 52, 6529–6535 (2011). 21715348 10.1167/iovs.11-7454 PMC3176031

[b13] Chong E. M. & Dana M. R. Graft failure IV. Immunologic mechanisms of corneal transplant rejection. Int Ophthalmol 28, 209–222 (2008). 17673946 10.1007/s10792-007-9099-9

[b14] Cursiefen C., Chen L., Dana M. R. & Streilein J. W. Corneal lymphangiogenesis: evidence, mechanisms, and implications for corneal transplant immunology. Cornea 22, 273–281 (2003). 12658100 10.1097/00003226-200304000-00021

[b15] Folkman J. Tumor angiogenesis: therapeutic implications. N Engl J Med 285, 1182–1186 (1971). 4938153 10.1056/NEJM197111182852108

[b16] Chen L. . Vascular endothelial growth factor receptor-3 mediates induction of corneal alloimmunity. Nature medicine 10, 813–815 (2004). 10.1038/nm1078 15235599

[b17] Dashkevich A. . Lymph angiogenesis after lung transplantation and relation to acute organ rejection in humans. Ann Thorac Surg 90, 406–411 (2010). 20667320 10.1016/j.athoracsur.2010.03.013

[b18] Kerjaschki D. . Lymphatic endothelial progenitor cells contribute to de novo lymphangiogenesis in human renal transplants. Nature medicine 12, 230–234 (2006). 10.1038/nm1340 16415878

[b19] Stuht S. . Lymphatic neoangiogenesis in human renal allografts: results from sequential protocol biopsies. Am J Transplant 7, 377–384 (2007). 17283487 10.1111/j.1600-6143.2006.01638.x

[b20] Geissler H. J. . First year changes of myocardial lymphatic endothelial markers in heart transplant recipients. Eur J Cardiothorac Surg 29, 767–771 (2006). 16439147 10.1016/j.ejcts.2005.12.024

[b21] Grimaldo S., Yuen D., Ecoiffier T. & Chen L. Very late antigen-1 mediates corneal lymphangiogenesis. Investigative ophthalmology & visual science 52, 4808–4812 (2011). 21372020 10.1167/iovs.10-6580 PMC3175962

[b22] Choi I. . 9-cis retinoic acid promotes lymphangiogenesis and enhances lymphatic vessel regeneration: therapeutic implications of 9-cis retinoic acid for secondary lymphedema. Circulation 125, 872–882 (2012). 22275501 10.1161/CIRCULATIONAHA.111.030296 PMC3327127

