# Supplementary material for: Intravital Imaging Reveals Dynamics of Lymphangiogenesis and Valvulogenesis
Source: Sci Rep. 2016 Jan 20;6:19459. doi: 10.1038/srep19459 (PMC4726360; doi:10.1038/srep19459)
Supplement: Supplementary Information [file srep19459-s1.doc]

**Intravital Imaging Reveals Dynamics of Lymphangiogenesis and Valvulogenesis**

Gyeong J. Kang^1,2^, Tatiana Ecoiffier^1,2^, Tan Truong^1,2^, Don Yuen^1,2^, Guangyu Li^1,2^, Narae Lee^1,2^, Liwei Zhang^1,2^, Lu Chen^1, 2*^

^1^Vision Science Graduate Group, University of California, Berkeley, CA 94720, USA; ^2^Center for Eye Disease and Development, Program in Vision Science, and School of Optometry, University of California, Berkeley, CA 94720, USA

**Supplementary Video 1.** Intravital video showing lateral migration of endothelial stalk cells (indicated by magenta arrows) along the axis of lymphatic vessel elongation.
